# Supplementary material for: Causal Relationship between Aging and Anorexia Nervosa: A White-Matter-Microstructure-Mediated Mendelian Randomization Analysis
Source: Biomedicines. 2024 Aug 16;12(8):1874. doi: 10.3390/biomedicines12081874 (PMC11351342; doi:10.3390/biomedicines12081874)
Supplement: Supplementary file 1 [file biomedicines-12-01874-s001.zip › Supplementary Figure/Figure S8/Leaveoneout plot of MR analysis for TL to ubm-b-1466.pdf]

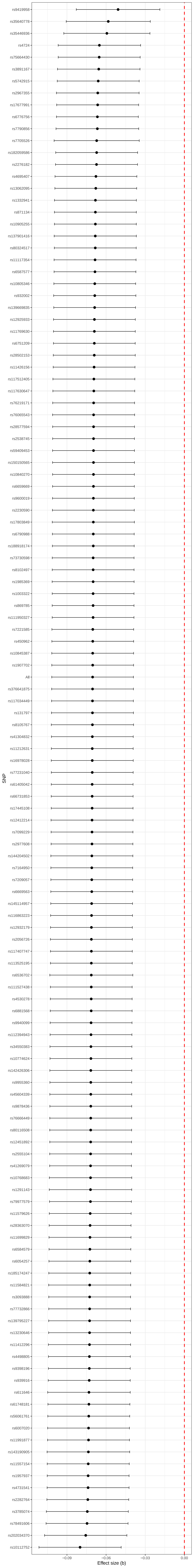
rs9419958
rs35640778
rs35446936
rs4724
rs75664430
rs3891167
rs5742915
rs2967355
rs17677991
rs6776756
rs7790856
rs7705526
rs182059586
rs2276182
rs4695407
rs13062095
rs1332941
rs871134
rs10905255
rs137901416
rs80324517
rs11117354
rs6587577
rs10805346
rs932002
rs139669835
rs12925933
rs11769630
rs6751209
rs28502153
rs11426156
rs117512405
rs117630647
rs76219171
rs76065543
rs28577594
rs2538745
rs59409453
rs150150565
rs10840270
rs6659669
rs9600019
rs2230590
rs17803849
rs6790988
rs188918174
rs73730598
rs8102497
rs1985369
rs1003322
rs869785
rs111950327
rs7221585
rs450962
rs10845387
rs1907702
All
rs376641875
rs117034449
rs131797
rs8105767
rs41304832
rs11212631
rs16978028
rs77231040
rs61405042
rs66731853
rs17445108
rs12412214
rs7099229
rs2977608
rs144204502
rs7164950
rs7209057
rs6669563
rs145114957
rs116863223
rs12932179
rs2056726
rs117407747
rs113525195
rs6536702
rs111527438
rs4530278
rs6881568
rs9940099
rs112394943
rs34550383
rs10774624
rs142426306
rs9955360
rs45604339
rs9878436
rs76666449
rs80116508
rs12451892
rs2555104
rs41269079
rs10768683
rs1291143
rs79977579
rs11579626
rs28363070
rs11699829
rs6584579
rs6054257
rs185174247
rs11584821
rs3093888
rs77732866
rs139795227
rs13230646
rs11412296
rs4498805
rs9398196
rs939916
rs611646
rs61748181
rs56061761
rs6007020
rs11991877
rs143190905
rs11557154
rs1957937
rs4731541
rs2282764
rs3785074
rs78491606
rs202034370
rs10112752
SNP
-0.09
-0.06
-0.03
0.00
Effect size (b)
